# Xanthine oxidase inhibitory and antioxidant potential of Indian *Muscodor* species

**DOI:** 10.1007/s13205-016-0569-5

**Published:** 2016-11-17

**Authors:** Neha Kapoor, Sanjai Saxena

**Affiliations:** Department of Biotechnology, Thapar University, Patiala, Punjab 147004 India

**Keywords:** Endophytic fungi, DPPH assay, Enzyme inhibitor, Muscodor, Hyperuricemia

## Abstract

Xanthine oxidase is a key enzyme responsible for hyperuricemia, a pre-disposing factor for Gout and oxidative stress-related diseases. Only two clinically approved xanthine oxidase inhibitors Allopurinol and Febuxostat are currently used for treatment of hyperuricemia. However, owing to their side effects there is a need for new non-purine-based selective inhibitors of xanthine oxidase. In the process of exploring novel xanthine oxidase inhibitors and anti-oxidants, we screened the culture filtrate of 07 novel species of *Muscodor*, a sterile endophytic fungi isolated from *Cinnamomum* and *Aegle marmelos*. Chloroform extract of *M. darjeelingensis* exhibited the maximum xanthine oxidase inhibition in the qualitative and quantitative assays. The IC_50_ of chloroform extract of *M. darjeelingensis* was 0.54 µg/ml which was much lower to Allopurinol but higher when compared to Febuxostat. 88% reduction in uric acid production was recorded *by M. darjeelingensis* chloroform extract which was similar to allopurinol. The maximum anti-oxidant activity was exhibited by *M. indica* against the gallic acid standard in the DPPH-free radical assay. Anti-oxidant activity index of *M. indica* was 7.7, which was followed by *M. kashayum* with 5.4. *M. darjeelingensis* exhibited a moderate anti-oxidant activity with anti-oxidant activity index of 1.63 in the DPPH assay. The present study is the very first report of *Muscodor* species exhibiting xanthine oxidase inhibitory and anti-oxidant activity together. Chloroform extract of *M. darjeelingensis* and *M. indica* stand out as potential candidates for isolation and characterization of the xanthine oxidase inhibitor and anti-oxidant compound, respectively.

## Introduction

Hyperuricemia is a pre-disposing factor of gout which has been recognized as a lifestyle disease affecting adult population in the developed as well as developing countries (Kuo et al. 2015). Hyperuricemia results due to high serum urate levels which is attributed to it’s over production or under-excretion. The therapeutic strategies of treatment of hyperuricemia are by excretion of excessive uric acid or blocking the uric acid production. The later strategy appears to be safer since it involves the inhibition of Xanthine oxidase (XO), the key enzyme responsible for the production of uric acid. Till date, only Allopurinol and Febuxostat have been clinically approved as XO inhibitors for the treatment of hyperuricemia and gout. However, there is a demand for new non-purine-based selective inhibitors of Xanthine oxidase (NP-SIXO’s) owing to the side effects of currently used drugs.

Over last two decades, endophytic fungi have been well recognized as fountainheads of novel bioactive compounds possessing anti-cancer, anti-microbial, and anti-oxidant properties as well as putative sources of phytochemicals. However, exploration of these microorganisms for NP-SIXO’s is very limited as evident from the literature (Kapoor and Saxena 2014).

The genus *Muscodo*r emerged with the discovery of sterile endophytic fungus *Muscodor albus* from the branch of cinnamon plant in Honduras (Worapong et al. 2001). Since then over 19 species have been added to the genus *Muscodor* on the basis of morphological, volatile gas composition, phenetic, and genetic makeup from Central/South America, Northern Territory of Australia, Thailand, China, and India (Meshram et al. 2014; Saxena et al. 2014a).

Till date, only volatile organic compounds (VOCs) produced by *Muscodor* have been explored and exploited for their antimicrobial, anti-insecticidal, and anti-fungal properties (Newman and Cragg 2015; Saxena et al. 2014b). Secondary metabolites of *Muscodor* species have not been explored extensively; there is only a single report on antimicrobial activity (Boparai et al. 2015). Hence, *Muscodor* species can be a novel source of new and diverse bioactive moieties which could be exploited by the pharmaceutical and the agrochemical industry.

Thus, in the present investigation, we have evaluated the in vitro xanthine oxidase inhibitory and antioxidant potential of non-volatile secondary metabolites of Indian *Muscodor* species.

## Materials and methods

### Production of secondary metabolites

Indian *Muscodor* species viz. *Muscodor strobelii*, *M. darjeelingensis*, *M. tigerii*, *M. kashayum*, *M. ghoomensis*, *M. indica,* and *M. camphora* were inoculated in potato dextrose broth for secondary metabolite production. Briefly, 5 mm mycelial plug of 3–4 day-old culture was inoculated into 100 ml pre-sterilized Potato Dextrose Broth (pH 5.1) followed by incubation at 26 ± 2 °C, 120 rpm for 7 days. Subsequently, the fungal mass was separated by filtration through Whatman filter paper No. 4 followed by centrifugation at 10,000 rpm for 10 min. The supernatant so obtained was subjected to qualitative XOI assay.

### Qualitative screening of XO inhibition

Qualitative screening of XO inhibition was carried out as per the procedure of Kapoor and Saxena (2014). The method comprised of preparation of Xanthine–Nitroblue tetrazolium (NBT) plates using 0.8% agar, 1.5 mg/ml Xanthine, and 0.11 mg/ml NBT. 5 mm wells were prepared aseptically with a sterile cork borer. Subsequently, 40 μl of reaction mixture containing 30 μl of each culture filtrate, 0.04 U of xanthine oxidase (source: bovine milk), and 10 mmol/L of Tris–HCl buffer was dispensed into each well followed by overnight incubation at 37 °C. The control well consisted of 30 µl of un-inoculated broth and 0.04 U of XO. Allopurinol and Febuxostat (1 mM) were used as positive controls. Appearance of a blue-colored halo indicated the XO activity in control well while reduction in diameter of blue-colored halo in comparison to control-indicated XO inhibition. All the tests were carried out in triplicates. The halo diameter was recorded and data were represented as mean ± SD values.

### Metabolite extraction from the culture filtrates

The cell-free supernatant of each culture was extracted thrice with chloroform in the ratio of 1:2. The organic layers were pooled followed by dehydration with anhydrous sodium sulphate. The solvent was evaporated till dryness at room temperature so as to get chloroform fraction residue. The fraction so obtained was weighed and reconstituted in methanol.

### Quantitative estimation of xanthine oxidase inhibition

#### NBT assay

The crude chloroform fractions of cultures were subjected for determination of XOI as described by Aggarwal and Banerjee (2009) with slight modifications. The crude fractions were pre-incubated with bovine milk xanthine oxidase at 37 °C for 1 h prior to assay. The reaction was initiated by addition of 130 μL of xanthine (10 mM) followed by 30 μl of NBT. After the incubation, the amount of formazan formed was estimated by measuring the absorbance at 575 nm using a microplate reader (Biotek Powerwave 340, USA). Allopurinol and Febuxostat were used as positive control. Control reaction mixture consisted of substrate, enzyme, and NBT without any inhibitor. All the reactions were performed in triplicates.

#### Uric acid estimation assay

This assay was carried out as per the method of Chang et al. (1993), wherein the reaction mixture comprised of 10 µl of crude chloroform extract and 990 µl of xanthine buffer solution (200 µM). The reaction was initiated by addition of 5 µl of XO solution. Subsequently, the reaction mixture was mixed properly followed by incubation at 25 °C for 15 min. The reaction was terminated by adding 1 N HCl solution. Subsequently, the reaction was aborted by adding 1 N HCl solution. The concentration of uric acid was measured by taking absorbance value at 290 nm. Allopurinol and Febuxostat were used as positive controls. The percentage inhibition of xanthine oxidase was calculated by following formula:

$$\% \,{\text{Inhibition}} = \left[{(A - B){-}(C - D)} \right]/(A - B) \times 100 \% ,$$

where *A* is the OD at 290 nm with enzyme but without sample, *B* is the OD at 290 nm without sample and enzyme, *C* is the OD at 290 nm with sample and enzyme, and *D* is the OD at 290 nm with sample but without enzyme.

#### Free radical scavenging activity by DPPH assay

Antioxidant potential of the chloroform extracts of cultures were determined by the procedure of Ho et al. (2012) with minor modifications. Briefly, the reaction mixture comprised of 100 μl of sample extract (1 mg/ml) mixed with 100 µL DPPH solution (1, 1-diphenyl-2-picrylhydrazyl, Sigma, Final concentration = 4 μg/ml). Allopurinol and Febuxostat were also evaluated for their antioxidant potential. The control comprised of 100 μl of methanol and 100 μl of DPPH solution. The titer plate was then incubated at 25 °C for 30 min in dark. The sudden decrease in absorbance was measured at 517 nm and the DPPH scavenging activity was calculated using the following formula:

$${\text{DPPH scavenging activity (}}\% ) = [{\text{Acontrol}} - {\text{ATest}}]/{\text{Acontrol }} \times { 1}00.$$

The test was performed in triplicates and data were represented as mean ± SD. The antioxidant capacity was expressed as the antioxidant activity index (AAI) determined by following formula

AAI = final concentration of DPPH (μg/ml)/IC_50_ of sample (μg/ml).

An AAI of 0.5 and below indicated a poor anti-oxidant, when AAI was between 0.5 and 1.0 it is moderate anti-oxidant,and when the AAI is in the range of 1.0 and 2.0 it is considered as a strong anti-oxidant, while above 2 AAI indicated a very strong anti-oxidant (Scherer and Godoy 2009).

### Test for purine

The chloroform fraction of *Muscodor* species exhibiting the best XO inhibition was test for the presence of purine moieties using a silver precipitation test (Kapoor and Saxena 2014). Briefly the assay involved 0.5 ml of test sample (Stock-1 mg/ml) in test tube and excess of ammonium hydroxide was added to it followed by addition of 0.5 ml of 0.1 M silver nitrate solution. Appearance of white precipitate, which is Purine-Ag+ complex, indicates the presence of purine. Allopurinol (purine analogue) was used as positive control, and Febuxostat, being non-purine in nature, served as negative control.

## Results

### Qualitative XO inhibition assay

**Fig. 1:**
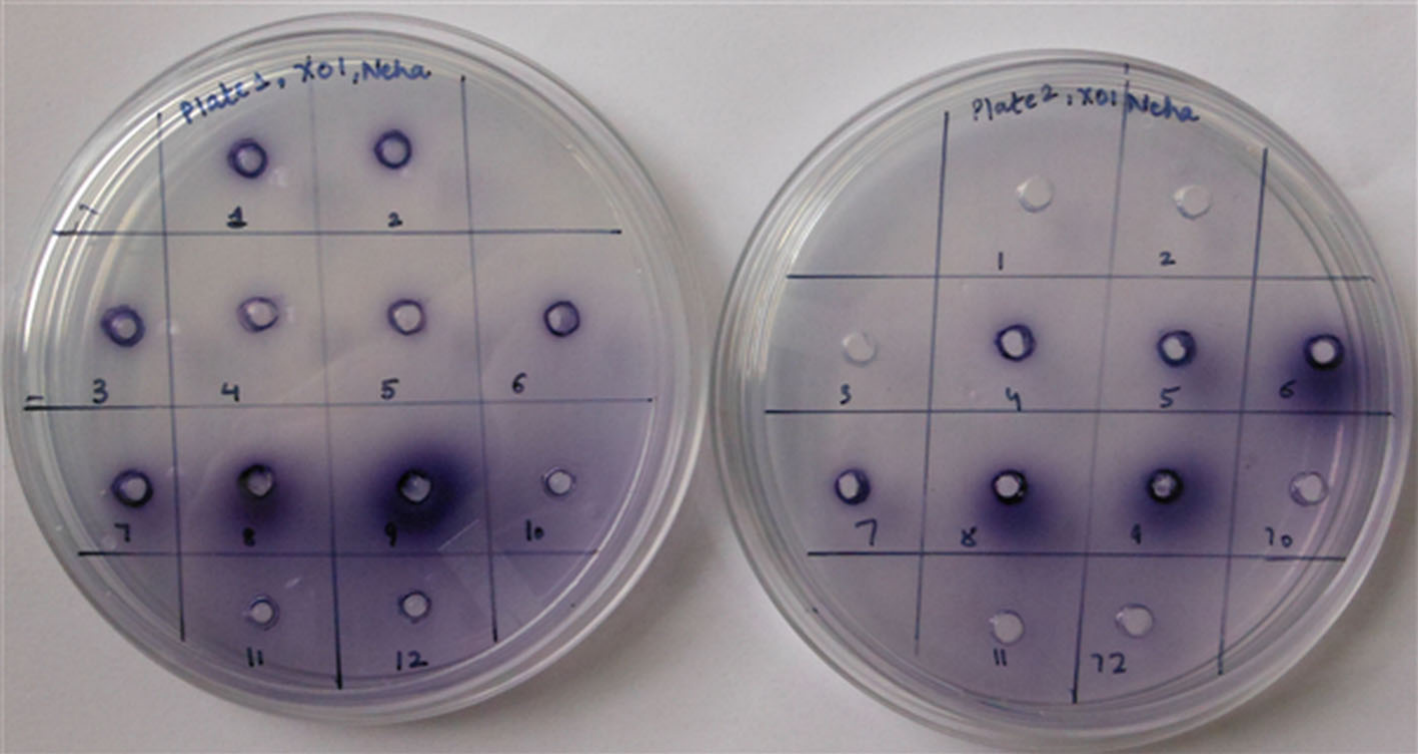
Qualitative NBT plate assay of chloroform residues of *Muscodor* species

*Muscodor darjeelingensis* chloroform extract (CE) exhibited the highest inhibition of XO (59.4%) all the *Muscodor* species tested. This was similar to the clinically approved first NP-SIXO, Febuxostat used as a standard. It was closely followed by *M. tigerii* with 56.7% inhibition which was marginally lower to allopurinol exhibiting 58.6% XO inhibition in the assay. In the qualitative assay, *M. indica* CE exhibited the least XO inhibition. Febuxostat, Allopurinol, *M. darjeelingensis* CE, *M. tigerii* CE, and *M. kashayum* CE exhibited a similar XO inhibition profile in the plate assay based on Tukey’s post hoc analysis (Table 1; Fig. 1).

**Table 1:** Xanthine oxidase inhibition of Indian *Muscodor* species by qualitative and quantitative assay

Treatment	% XO inhibition		% Reduction in uric acid production
	Xanthine plate assay	NBT assay	
Control (no inhibitor)	0^e^	0^g^	0^g^
Febuxostat*	59.4 ± 1.0^a^	99.2 ± 0.0^a^	99.5 ± 0.0^a^
*M. darjeelingensis*	59.4 ± 1.0^a^	91.4 ± 0.8^b^	88.1 ± 0.0^b^
Allopurinol*	58.6 ± 1.0^a^	88.0 ± 0.5^b^	86.7 ± 0.0^b^
*M. tigerii*	56.7 ± 0.9^a^	77.0 ± 0.5^c^	74.0 ± 0.0^c^
*M. kashayum*	54.9 ± 1.0^a^	76.0 ± 1.0^c^	70.3 ± 0.0^c^
*M. strobelii*	48.6 ± 3.1^b^	40.2 ± 1.9^e^	40.2 ± 2.6^d^
*M. camphora*	46.7 ± 1.8^b^	46.7 ± 1.8^d^	39.7 ± 0.0^d^
*M. ghoomensis*	31.6 ± 3.8^c^	37.6 ± 2.7^e^	24.2 ± 0.0^e^
*M. indica*	18.2 ± 1.9^d^	28.6 ± 4.5^f^	18.7 ± 1.6^f^

* Represent commercial inhibitors of Xanthine Oxidase (XO). All values presented are Mean ± SD of triplicate readings

Mean values represented by same alphabets are not significantly different by Tukey’s post hoc analysis at *p* ≤ 0.05

### Quantitative XO inhibition

**Fig. 2:**
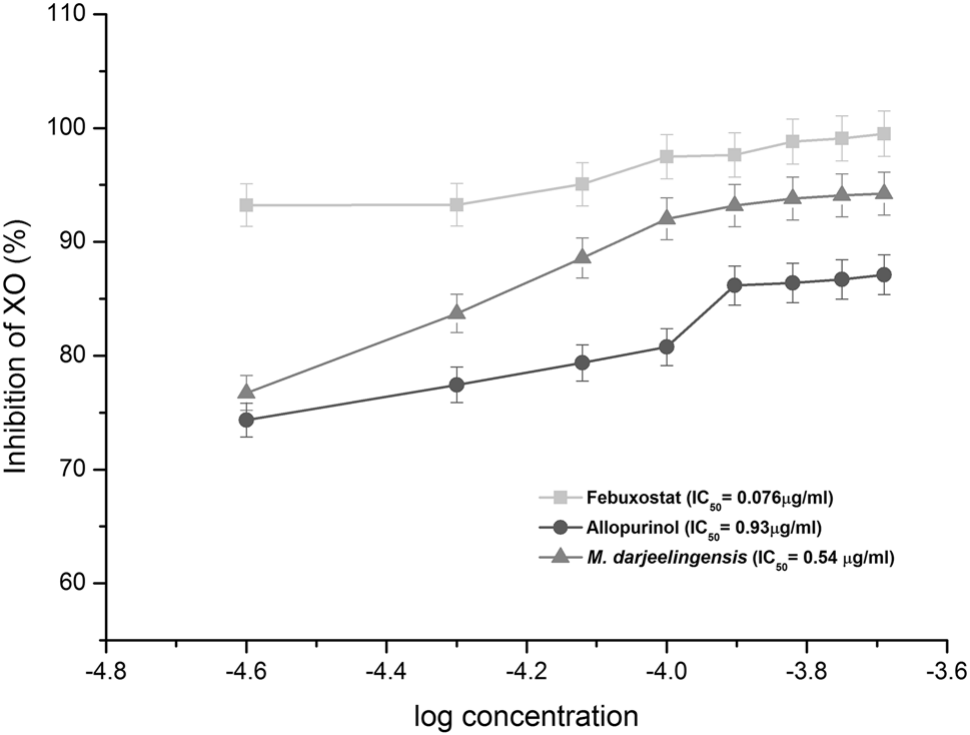
Dose-response curve of chloroform residue of *M. darjeelingensis* for inhibition of xanthine oxidase. Allopurinol and Febuxostat was used as the positive control

In the quantitative NBT-based XO inhibition assay, Febuxostat exhibits 99.2% inhibition of the XO activity which was closely followed by *M. darjeelingensis* with 91.4% inhibition. Allopurinol tested as a purine-based XO inhibitor exhibited only 88% reduction in the XO activity during the in vitro assay. *M. tigerii* and *M. kashayum* CE extracts exhibited a moderate inhibition of 77% and 76%, respectively. Tukey’s post hoc analysis suggested that *M. darjeelingensis* CE and Allopurinol possessed similar potency for XO inhibition. These findings corroborated with the reduction in uric acid production, with maximum reduction in Febuxostat, followed by *M. darjeelingensis*, Allopurinol, and *M. tigerii* (Table 1). Further, *M. darjeelingensis* CE exhibited an IC_50_ value of 0.54 µg/ml for XO in the in vitro assay (Fig. 2).

### Free radical scavenging assay

**Fig. 3:**
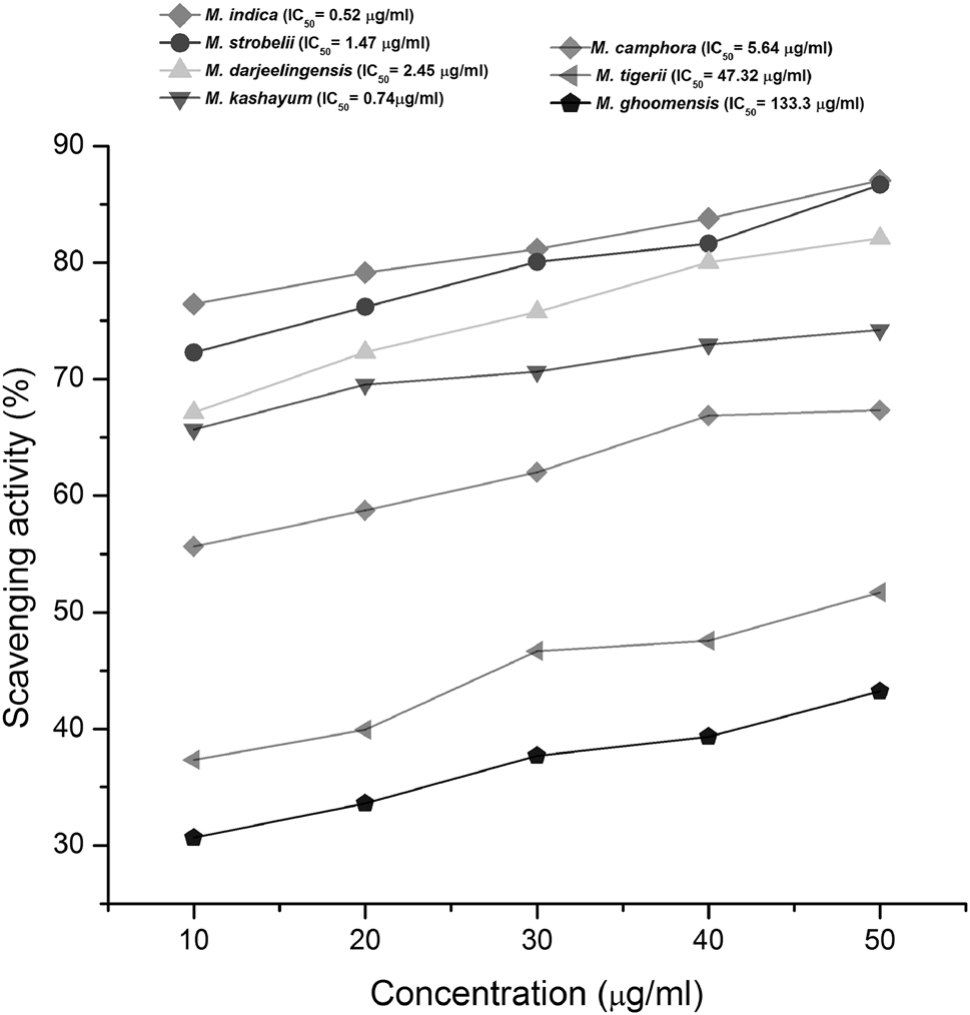
Dose-response curve of chloroform residue of Indian *Muscodor* species for antioxidant activity by DPPH assay

*Muscodor indica* CE exhibited the highest free radical scavenging activity among the isolates tested and was quite similar to the Gallic acid, the standard anti-oxidant used in the DPPH assay. The IC_50_ value was 0.5 µg/ml for *M. indica* CE as compared to 7 µg/ml of Gallic acid. It also exhibited the highest AAI of 7.7 as compared to Gallic acid (Table 2; Fig. 3). *M. ghoomensis* CE exhibited the least antioxidant activity as well as AAI.

**Table 2:** Free radical scavenging activity by DPPH Assay

Species	Scavenging activity (%)	IC_50_ (μg/ml)	Antioxidant activity index (AAI)
Gallic acid (standard)*	73.9 ± 1.3^a^	7.00	0.6
*M. indica*	71.6 ± 0.9^ab^	0.52	7.7
*M. strobelii*	69.8 ± 2.6^abc^	1.47	2.7
*M. darjeelingensis*	68.0 ± 3.8^bc^	2.45	1.6
*M. kashayum*	65.4 ± 3.8^c^	0.74	5.4
*M. camphora*	48.5 ± 3.2^d^	5.64	0.7
*M. tigerii*	46.5 ± 1.7^d^	47.32	0.08
*M. ghoomensis*	46.4 ± 4.9^d^	133.30	0.03

* Represent commercial inhibitors of Xanthine Oxidase (XO). All values presented are Mean ± SD of triplicate readings

Mean values represented by same alphabets are not significantly different by Tukey’s post hoc analysis at *p* ≤ 0.05

### Purine detection test

*Muscodor darjeelingensis* CE did not exhibit presence of any purine moiety in the purine detection test. Febuxostat also gave a negative purine test indicating that it is a NP-SIXO while Allopurinol was found to be positive for purine.

## Discussion and conclusion

Hyperuricemia is a biochemical abnormality which results in gout apart from oxidative stress-related diseases. Hence, the lowering plasma uric acid levels within normal range is extremely important and can be achieved by blocking the uric acid production. Till date, only Allopurinol and Febuxostat have been clinically used for the treatment of hyperuricemia and gout; however, they have severe side effects which demand exploration of new XO inhibitors which are non- purine in nature and have lesser side effects as compared to synthetic chemicals. Endophytic fungi are relatively under tapped resources of XO inhibitors which could enter the drug discovery pipeline as anti-hyperuricemic agents. In the present study, CE of *M. darjeelingensis* was found to have a significantly lower IC_50_ value as compared to Fusaruside and Phenolic compounds isolated from endophytic *Fusarium* sp. IFB-121 and *Chaetomium* sp., respectively (Shu et al. 2004; Huang et al. 2007). Further phytochemicals like Isoliquiritigenin, Liquiritigenin, and Cinnamaldehyde exhibited an IC_50_ of 12.6, 14.29 and 8.4 µg/ml, respectively, which was much higher than 0.54 µg/ml of CE of *Muscodor darjeelingensis* (Wang et al. 2008). Earlier, we have reported that CE of another endophytic fungus *Lasiodiploda pseudotheobromae* exhibited a potent XO inhibition with an IC_50_ of 0.61 µg/ml (Kapoor and Saxena 2014); however, the CE of *M. darjeelingensis* exhibits a still lower IC_50_ for XO inhibition warranting potential for isolation and characterization of the bioactive moiety.

The XO inhibitory activity by endophytic fungi probably is attributed to their survival strategy to overcome the metabolically aggressive environment inside the plant. Further, they may also exhibit anti-oxidant potential to overcome the oxidative stress based defense mechanism of plants. Hence, we found that *M. indica* expressed highest anti-oxidant activity index when compared to the standard anti-oxidant Gallic acid. *Muscodor tigerii* and *M. ghoomensis* exhibited the least anti-oxidant activity index when compared to gallic acid. The IC_50_ of DPPH scavenging activity of *M. indica* was significantly low when compared to endophytic fungus *Cladosporium velox* TN-9S isolated from *T. cordifolia* (Singh et al. 2016). Endophytic fungi are increasingly being explored for their anti-oxidant activity; however, they are seldom being reported for both anti-oxidant and Xanthine oxidase inhibitory activity which are interconnected. Hence, in the present investigation, we have tried to establish both XO inhibitory as well as anti-oxidant activity of *Muscodor* species isolated from India primarily from *Cinnamomum* sp. and *Aegle marmelos*. Both *Cinnamomum* and *Aegle marmelos* are medicinal plants which have been previously reported to possess anti-oxidant potential (Upadhya et al. 2004; Mathew and Abraham 2006; Jayaprakasha et al. 2006; Prasad et al. 2009; Reddy and Urooj 2013). Further, it can be hypothesized that endophytes generally mimic the medicinal properties of their host.

Thus, it can be concluded from the present study that *M. darjeelingensis* possesses a potent XOI activity while *M. indica* possesses potent anti-oxidant activity which warrants further investigation for further isolation and characterization of bioactive compounds.
